# Practice variation and outcomes of minimally invasive *minor* liver resections in patients with colorectal liver metastases: a population-based study

**DOI:** 10.1007/s00464-023-10010-3

**Published:** 2023-04-18

**Authors:** Michelle R. de Graaff, Joost M. Klaase, Ruben de Kleine, Arthur K. E. Elfrink, Rutger-Jan Swijnenburg, Babs M. Zonderhuis, J. Sven D. Mieog, Wouter J. M. Derksen, Jeroen Hagendoorn, Peter B. van den Boezem, Arjen M. Rijken, Paul D. Gobardhan, Hendrik A. Marsman, Mike S. L. Liem, Wouter K. G. Leclercq, Tjarda N. T. van Heek, Gijs A. Pantijn, Koop Bosscha, Eric J. T. Belt, Maarten Vermaas, Hans Torrenga, Eric R. Manusama, Petrousjka van den Tol, Steven J. Oosterling, Marcel den Dulk, Dirk J. Grünhagen, Niels F. M. Kok, For Dutch Hepato Biliary Audit Group, Collaborators

**Affiliations:** 1 Dutch Institute for Clinical Auditing, Scientific Bureau, 2333 AA Leiden, The Netherlands; 2 Department of Hepato-Pancreato-Biliary Surgery and Liver Transplantation, University Medical Centre Groningen, Groningen, The Netherlands; 3 Department of Surgery, Cancer Centre Amsterdam, Amsterdam UMC, University of Amsterdam, Amsterdam, The Netherlands; 4 Department of Surgery, Leiden University Medical Centre, Leiden, The Netherlands; 5 Department of Surgery, University Medical Centre Utrecht, Utrecht, The Netherlands; 6 Department of Surgery, St Antonius Hospital, Nieuwegein, The Netherlands; 7 Department of Surgery, Radboud Medical Centre, Nijmegen, The Netherlands; 8 Department of Surgery, Amphia Medical Centre, Breda, The Netherlands; 9 Department of Surgery, OLVG, Amsterdam, The Netherlands; 10 Department of Surgery, Medical Spectrum Twente, Enschede, The Netherlands; 11 Department of Surgery, Máxima Medical Centre, Veldhoven, The Netherlands; 12 Department of Surgery, Gelderse Vallei, Ede, The Netherlands; 13 Department of Surgery, Isala, Zwolle, The Netherlands; 14 Department of Surgery, Jeroen Bosch Hospital, ‘s Hertogenbosch, The Netherlands; 15 Department of Surgery, Albert Schweitzer Hospital, Dordrecht, The Netherlands; 16 Department of Surgery, Ijsselland Hospital, Capelle Aan de Ijssel, The Netherlands; 17 Department of Surgery, Deventer Hospital, Deventer, The Netherlands; 18 Department of Surgery, Medical Centre Leeuwarden, Leeuwarden, The Netherlands; 19 Department of Surgery, Spaarne Gasthuis, Haarlem/Hoofddorp, The Netherlands; 20 Department of Surgery, Maastricht University Medical Centre, Maastricht, The Netherlands; 21 Department of Surgical Oncology, Erasmus MC Cancer Institute, Rotterdam, The Netherlands; 22 Department of Surgery, Antoni van Leeuwenhoek – Dutch Cancer Institute, Amsterdam, The Netherlands

**Keywords:** Minimally invasive liver surgery, Colorectal liver metastases, Hospital variation, Minor liver resection, Overall survival, Short-term outcomes

## Abstract

**Introduction:**

In 2017, the Southampton guideline stated that minimally invasive liver resections (MILR) should considered standard practice for minor liver resections. This study aimed to assess recent implementation rates of minor MILR, factors associated with performing MILR, hospital variation, and outcomes in patients with colorectal liver metastases (CRLM).

**Methods:**

This population-based study included all patients who underwent minor liver resection for CRLM in the Netherlands between 2014 and 2021. Factors associated with MILR and nationwide hospital variation were assessed using multilevel multivariable logistic regression. Propensity-score matching (PSM) was applied to compare outcomes between minor MILR and minor open liver resections. Overall survival (OS) was assessed with Kaplan–Meier analysis on patients operated until 2018.

**Results:**

Of 4,488 patients included, 1,695 (37.8%) underwent MILR. PSM resulted in 1,338 patients in each group. Implementation of MILR increased to 51.2% in 2021. Factors associated with not performing MILR included treatment with preoperative chemotherapy (aOR 0.61 CI:0.50–0.75, *p* < 0.001), treatment in a tertiary referral hospital (aOR 0.57 CI:0.50–0.67, *p* < 0.001), and larger diameter and number of CRLM. Significant hospital variation was observed in use of MILR (7.5% to 93.0%). After case-mix correction, six hospitals performed fewer, and six hospitals performed more MILRs than expected. In the PSM cohort, MILR was associated with a decrease in blood loss (aOR 0.99 CI:0.99–0.99, *p* < 0.01), cardiac complications (aOR 0.29, CI:0.10–0.70, *p* = 0.009), IC admissions (aOR 0.66, CI:0.50–0.89, *p* = 0.005), and shorter hospital stay (aOR CI:0.94–0.99, *p* < 0.01). Five-year OS rates for MILR and OLR were 53.7% versus 48.6%, *p* = 0.21.

**Conclusion:**

Although uptake of MILR is increasing in the Netherlands, significant hospital variation remains. MILR benefits short-term outcomes, while overall survival is comparable to open liver surgery.

**Graphical abstract:**

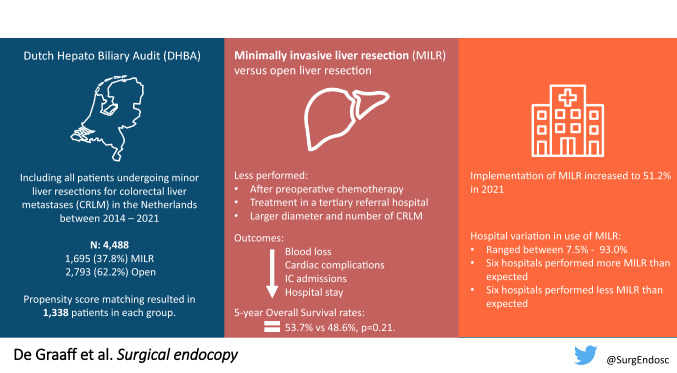

**Supplementary Information:**

The online version contains supplementary material available at 10.1007/s00464-023-10010-3.

Liver resection is a fundamental aspect of the curative treatment of patients with colorectal liver metastases (CRLM). In the past decades liver surgery has progressively changed by the introduction of laparoscopic and robotic liver resection.

Several meta-analyses compared minimally invasive liver resections (MILR) with open liver resection (OLR). These studies demonstrated superior short-term outcomes for MILR, including reduced blood loss, shorter hospital stay, reduced overall morbidity, and reduced major morbidity [1, 2]. Furthermore, mortality was not different between MILR and OLR, nor were oncologic outcomes significantly different between the two approaches [1–3]. Based on these results, the Southampton guidelines (2017) stated that a laparoscopic approach should be considered the standard practice for left lateral and anterior segments, primarily minor liver resections (i.e., resection of less than three adjacent liver segments) [4].

Before enactment of this Southampton guideline, the uptake of MILR in the Netherlands was relatively slow, with an implementation rate of 23% in 2016 [5]. Insight into hospital variation and factors associated with performance of MILR might provide guidance to enhance the uptake of MILR. Nationwide hospital variation, contributing factors and implementation after publication of the guideline have not been described.

This nationwide, population-based study aimed to assess current implementation rates of MILR for minor liver resections, as well as factors associated with performing MILR and with hospital variation. In addition, short- and long-term outcomes were assessed.

## Methods

### Study design

This nationwide, population-based study was performed with data retrieved from the Dutch Hepatobiliary Audit (DHBA). The DHBA is a mandatory audit in which all patients who underwent liver resection and/or thermal ablation in the Netherlands were registered [6]. Data verification in 2017 showed data completeness of 97% [7]. Data are handled anonymously; therefore, according to Dutch Law, no ethical approval is needed. The DHBA scientific committee approved the current study protocol.

Liver surgery is not performed in all hospitals in the Netherlands. Since the start of the DHBA, a required minimal volume of 20 liver resections per year (any resection, any surgical approach, any indication), has led to centralisation of liver surgery in the Netherlands [8]. Oncological networks are hospitals with referral pattern agreements, with at least one tertiary referral centre and multiple regional hospitals [9].

### Patient selection

This study included all patients of 18 years and older who are registered in the DHBA with CRLM and who underwent a liver resection between January 1, 2014, and December 31, 2021. Patients were excluded when essential data were missing (date of birth, indication for surgery or surgical approach, extent of resection). Patients were excluded from analysis when they underwent major liver resection (resection of ≥ 3 adjacent Couinaud segments) [10], repeat liver resection, two-stage liver resection, or thermal ablation alone.

### Combined datasets

For the assessment of long-term survival, DHBA data were linked to data from the Dutch national claim database for health insurance (Vektis) [11]. This database included the date of death because health care insurance ends when a Dutch citizen deceases. Since health care insurance is obligatory, it covers 99% of all Dutch inhabitants. Datasets were combined based on personal citizen numbers. From 2014 until 2018, 92.6% of both datasets could be linked. Since the introduction of the General Data Protection Regulation (GDPR) in 2018, some hospitals stopped registering these personal citizen numbers [12]. As a result, the linkage percentage between both datasets declined to 57.8% between 2019 and 2021. Therefore, overall survival was assessed using a subset of patients who underwent minor liver resections between 2014 and 2018. Datasets were merged in May 2022, and patients without a date of death were assumed to be alive.

### Operative procedure

For analysis, patients were divided into two treatment groups: patients who received an open minor liver resection and a minor MILR. MILR was defined as either a laparoscopic or robotic liver resection, as these two techniques were separated into two different categories in the DHBA from 2020 onwards. The operative approach was categorised by intention-to-treat.

In the DHBA, minor liver resections are defined as resection of ≤ 2 adjacent Couinaud segments.

#### Variables for analysis

Patient characteristics used for analysis included sex, age, body mass index (BMI), Charlson Comorbidity Index (CCI), and American Society of Anaesthesiologists (ASA) grade. Tumour characteristics used for analysis included number of CRLM, diameter of the largest CRLM before tumour-specific treatment, synchronous or metachronous metastases, location of the primary tumour (rectal/colon), bilobar disease, and extrahepatic disease. Treatment characteristics used for analysis included type of treatment (resection or combined resection and thermal ablation), administration of preoperative chemotherapy, and type of hospital where treatment took place (tertiary referral centre or regional hospital). The location of the metastasis per liver segment was available from 2020.

### Outcomes

Implementation of MILR was compared between hospitals and displayed as proportion of MILR of the total performed minor liver resections. The annual number of hospitals performing liver surgery was assessed. Perioperative outcomes included blood loss during resection. Outcomes after surgery included data on 30-day overall morbidity, 30-day major morbidity, in-hospital or 30-day mortality, length of hospital stay, postoperative ICU admission, resection margins after surgery, and overall survival.

Perioperative blood loss was measured in millilitres. 30-day major morbidity was defined as complications grade 3a or higher according to the Clavien-Dindo classification [13]. Length of hospital stay was calculated from the day of surgery to the day of discharge. Resection margins were defined as R0 (tumour-free margin ≥ 1 mm), R1 (tumour-free margin < 1 mm), and R2 (macroscopic incomplete tumour resection). Overall survival was calculated as the number of days from date of surgery to the date of death from any cause.

### Statistical analysis

Baseline characteristics between groups were compared using the chi-square or Fisher exact test as appropriate for categorical variables. The independent students' *t*-test was used for continuous variables and was presented as mean with interquartile ranges (IQR).

Univariable and multivariable multilevel regression analyses were used to assess possible case-mix factors associated with performing MILR. Case-mix factors were defined as non-modifiable patient and tumour characteristics [14]. All possible case-mix factors were entered in the univariable regression analysis. If a significant association was found (*p* < 0.1, Wald test), the variable was entered into the multivariable model. Statistical significance was defined as a two-sided *p*-value < 0.05 in the multilevel multivariable model. Multilevel analysis was used to account for the year of surgery.

To compare the implementation of MILR, all hospitals that performed liver surgery upward from 2018 were considered. Hospital variation was assessed using case-mix corrections to correct for confounding factors associated with performing MILR and displayed using an observed/expected (O/E) ratio. Observed is the absolute number of MILRs performed per hospital and expected is the number of expected performed MILRs based on a multivariable multilevel logistic regression model which included case-mix variables. This results in a case-mix corrected variability in the use of MILR between hospitals. An O/E-ratio above 1 indicates a hospital performed more MILR than expected, and an O/E-ratio below 1 indicates a hospital performed less MILR than expected. Outliers were statistically significant if hospitals fell out of the 95% confidence intervals.

#### Propensity-score matching

To evaluate differences in postoperative outcomes between MILR and OLR, propensity score matching (PSM) was performed. Propensity scores were estimated using a multivariable logistic regression model. PSM was performed using a 1:1 ratio with the nearest neighbour method and a calliper of 0.05. Covariates used for PSM included age, ASA score, diameter of CRLM, number of CRLM, extrahepatic disease, and preoperative chemotherapy. Balancing after matching was tested using standardised mean differences (SMD). SMD < 0.1 indicated negligible differences between both groups.

After PSM, postoperative outcome variables were tested in a univariable regression model. If a significant association was found (*p* < 0.1, Wald test), the variable was entered into the multivariable model. Statistical significance was defined as a two-sided *p*-value < 0.05 in the multivariable model. Overall survival (OS) was assessed from date of surgery. Kaplan–Meier survival analyses with the log-rank test were used to compare OS between patients treated with MILR and OLR.

Multicollinearity was assessed with a variance inflation factor (VIF). Variables were considered multicollinear if VIF exceeded 2.5. All analyses were performed using R version 4.1.0 (R Core Team (2021). (R: A language and environment for statistical computing. R Foundation for Statistical Computing, Vienna, Austria).

## Results

### Baseline characteristics

A total of 4,488 patients were included in this study (Supplemental figure 1). Of all patients included, 1,695 patients (37.8%) underwent minor MILR, and 2,793 (62.2%) underwent minor OLR. Table 1 shows all baseline characteristics of patients undergoing MILR or OLR. Patients in the MILR group were older, had more comorbidities, and had higher ASA scores. Furthermore, they had fewer and smaller CRLM lesions and were less often diagnosed with bilobar disease, and more often with metachronous and extrahepatic disease. In the MILR group, patients were less often treated with preoperative chemotherapy or combined ablation, and treatment was less often in a tertiary referral hospital.

**Table 1:** Baseline characteristics and surgical approach minimally invasive minor liver resection (MILR) or open liver resection (OLR) of patients who underwent a liver resection for CRLM between 2014 and 2020 in the Netherlands

Factor	Before matching			After matching			
	MILR *N* = 1695 (%)	OLR *N* = 2793 (%)	*p*-value	MILR *N* = 1338	OLR *N* = 1338	*p*-value	SMD
Sex			0.73			0.14	0.08
Male	1073 (63.3)	1747 (62.5)		839 (62.7)	820 (61.3)		
Female	620 (36.6)	1034 (37.0)		497 (37.1)	510 (38.1)		
Missing	2 (0.1)	12 (0.4)		2 (0.1)	8 (0.6)		
Age (years)			**0.01**			0.57	0.05
< 50	106 (6.3)	196 (7.0)		68 (5.1)	69 (5.2)		
50–64	556 (32.8)	1018 (36.4)		441 (33.0)	448 (33.5)		
65–80	869 (51.3)	1361 (48.7)		697 (52.1)	710 (53.1)		
> 80	164 (9.7)	218 (7.8)		132 (9.9)	111 (8.3)		
Charlson Comorbidity Index			**0.03**			0.86	0.01
CCI 0/1	1202 (70.9)	2065 (73.9)		967 (72.3)	962 (71.9)		
CCI 2 +	493 (29.1)	728 (26.1)		371 (27.7)	376 (28.1)		
BMI*			0.22			0.86	0.01
Mean (SD)	26.5 (4.64)	26.3 (4.36)		26.48 (4.54)	26.44 (4.31)		
Missing	24 (1.4)	64 (2.3)					
ASA score*			** < 0.001**			0.58	0.02
ASA 1/2	1233 (72.7)	2157 (77.2)		1046 (78.2)	1033 (77.2)		
ASA 3 +	446 (26.3)	595 (21.3)		292 (21.8)	305 (22.8)		
Missing	16 (0.9)	41 (1.5)					
Liver disease			**0.002**			0.73	0.03
Normal liver	1192 (70.3)	1831 (65.6)		942 (70.4)	924 (69.1)		
Yes	315 (18.6)	606 (21.7)		269 (20.1)	278 (20.8)		
Missing	188 (11.1)	356 (12.7)		127 (9.5)	136 (10.2)		
Preoperative chemotherapy			** < 0.001**			0.64	0.04
Yes	218 (12.9)	848 (30.4)		197 (14.7)	182 (13.6)		
Missing	53 (3.1)	213 (7.6)		46 (3.4)	51 (3.8)		
Number of lesions			** < 0.001**			0.99	0.03
1	1123 (66.3)	1022 (36.6)		813 (60.8)	814 (60.8)		
2	331 (19.5)	616 (22.1)		305 (22.8)	300 (22.4)		
3	110 (6.5)	348 (12.5)		109 (8.1)	107 (8.0)		
4	45 (2.7)	233 (8.3)		44 (3.3)	46 (3.4)		
5	33 (1.9)	178 (6.4)		32 (2.4)	31 (2.3)		
> 5	35 (2.1)	323 (11.6)		35 (2.6)	40 (3.0)		
Missing	18 (1.1)	73 (2.6)					
Maximum diameter largest tumour (mm)			** < 0.001**			0.97	0.03
< 20	654 (38.6)	757 (27.1)		445 (33.3)	432 (32.3)		
20–34	595 (35.1)	1027 (36.8)		525 (39.2)	527 (3.4)		
35–54	228 (13.5)	517 (18.5)		213 (15.9)	220 (16.4)		
55–998	65 (3.8)	233 (8.3)		59 (4.4)	57 (4.3)		
Missing	153 (9.0)	259 (9.3)		96 (7.2)	102 (7.6)		
Bilobar disease			** < 0.001**			**< 0.01**	0.36
No	1083 (63.9)	945 (33.8)		833 (62.3)	604 (45.1)		
Yes	355 (20.9)	1375 (49.2)		323 (24.1)	423 (31.6)		
Missing	257 (15.2)	473 (16.9)		182 (13.6)	311 (23.2)		
Location of primary tumour			**0.001**			**0.01**	0.10
Colon	1048 (61.8)	1857 (66.5)		818 (61.1)	885 (66.1)		
Rectal	647 (38.2)	930 (33.3)		520 (38.9)	453 (33.9)		
Missing	0 (0)	6 (0.2)					
Timing of metastases			** < 0.001**			0.13	0.06
Metachronous	979 (57.8)	1433 (51.3)		765 (57.2)	805 (60.2)		
Synchronous	716 (42.2)	1360 (48.7)		573 (42.8)	533 (39.8)		
Missing							
Extrahepatic disease			**0.01**			0.26	0.05
No	1466 (86.5)	2340 (83.8)		1170 (87.4)	1190 (88.9)		
Yes	190 (11.2)	398 (14.3)		168 (12.6)	148 (11.1)		
Missing	39 (2.3)	55 (2.0)					
Resection margin			** < 0.001**			0.49	0.06
R0	1528 (90.1)	2414 (86.4)		1205 (90.1)	1198 (89.5)		
R1	143 (8.4)	297 (10.6)		112 (8.4)	108 (8.1)		
R2	8 (0.5)	34 (1.2)		7 (0.5)	10 (0.7)		
Missing	16 (0.9)	48 (1.7)		14 (1.0)	22 (1.6)		
Ablation			** < 0.001**			** < 0.01**	0.25
No	1535 (90.6)	1916 (68.6)		1193 (89.2)	1072 (80.1)		
Yes	160 (9.4)	877 (31.4)		145 (10.8)	266 (19.9)		
Conversion rate							
Yes	235 (13.9)	–					
Type of hospital*			** < 0.001**			** < 0.01**	0.19
Other hospitals	1129 (66.6)	1408 (50.4)		886 (66.2)	760 (56.8)		
Tertiary Centres	566 (33.4)	1385 (49.6)		452 (33.8)	578 (43.2)		

Bold p-values indicate statistical signficance (p < 0.05)

^*^Abbreviations: MILR minimally invasive liver resection; OLR open liver resection; SMD standardized mean difference; BMI indicates body mass index; ASA score indicates American Association of Anaesthesiologist; type of hospital tertiary centre indicates hospitals with the highest expertise on oncological surgery

### Implementation of MILR

**Fig. 1:**
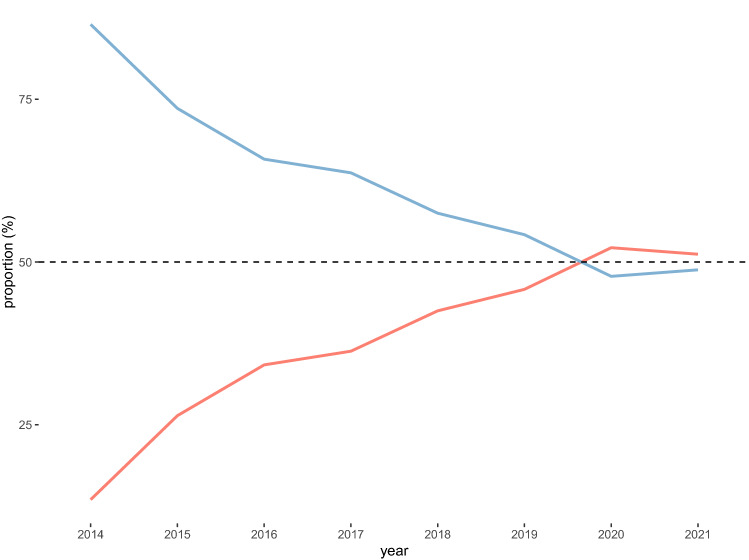
Proportion of performed MILR and OLR minor liver resection for CRLM between 2014–2021 in the Netherlands. Blue = Open liver resection (OLR). Orange = Minimally invasive liver resection (MILR). The dashed line represents 50% of all performed minor liver resections per year (Color figure online)

Since the start of the DHBA, the use of MILR increased from 13.5% in 2014 to 51.2% in 2021 (Fig. 1). In 2014, 26 hospitals performed a median of 15 (IQR 12–24) minor liver resections per year. In 2021, 22 hospitals performed a median of 24.5 (IQR 17–30.7) minor liver resections per year (Supplemental figure 2)*.*

### Factors associated with a minimally invasive liver resection

In multilevel multivariable analysis, nested for the year of surgery per individual, two factors were associated with increased use of MILR. These factors included synchronous disease (aOR 1.23 CI: 1.06–1.43, *p* = *0.007*) and primary tumour located in rectum (aOR 1.39 CI: 1.20–1.61, *p* < *0.001)* (Table 2). Four factors were associated with decreased use of MILR. These factors included treatment with preoperative chemotherapy (aOR 0.61 CI: 0.50–0.75, *p* < 0.001), treatment in a tertiary referral hospital (aOR 0.57 CI: 0.50–0.67, *p* < *0.001*), larger diameter and number of CRLM (Table 2). Multicollinearity was not observed.

**Table 2:** Univariable and multivariable logistic regression model of patient and tumour characteristics associated with performance of MILR minor resections in patients who underwent liver resection for CRLM between 2014 and 2021 in the Netherlands. Multilevel logistic regression model with individuals nested for year of surgery

Factor	Univariable				Multivariable		
	*N*	OR	95% CI	*p*-value	aOR	95% CI	*p*-value
Sex				0.71			
Male	2820	1					
Female	1654	0.97	0.86–1.10				
Missing*	14						
Age (years)				0.01			0.63
< 50	302	1			1		
50–64	1574	1.01	0.78–1.31	0.94	0.83	0.61–1.12	0.22
65–80	2230	1.18	0.92–1.52	0.19	0.89	0.66–1.20	0.44
> 80	382	1.39	1.01–1.90	0.03	0.87	0.59–1.24	0.41
Charlson Comorbidity Index (CCI)				0.02			0.39
CCI 0/1	3267	1			1		
CCI 2 +	1221	1.16	1.01–1.33		1.07	0.91–1.26	
BMI**				0.22			
Mean (SD)		1.01	0.99–1.02				
Missing							
ASA score*				< 0.001			0.22
ASA 1/2	3390	1			1		
ASA 3 +	1041	1.31	1.13–1.51		1.11	0.94–1.32	
Missing*	57						
Histopathology liver parenchyma £				0.004			0.81
Normal liver	3023	1			1	1	
Liver disease	921	0.79	0.68–0.93	0.004	1.06	0.88–1.27	0.55
Missing	544	0.81	0.66–0.98	0.03	1.98	0.77–1.27	0.84
Preoperative chemotherapy				< 0.001			** < 0.001**
No	3156	1			1		
Yes	1066	0.31	0.26–0.36	< 0.001	0.61	0.50–0.75	** < 0.001**
Missing	266	0.30	0.22–0.40	< 0.001	0.42	0.29–0.59	** < 0.001**
Number of lesions				< 0.001			** < 0.001**
1	2145	1			1	1	
2	947	0.48	0.41–0.57	< 0.001	0.56	0.46–0.68	** < 0.001**
3	458	0.28	0.22–0.36	< 0.001	0.34	0.26–0.45	** < 0.001**
4	278	0.17	0.12–0.24	< 0.001	0.22	0.15–0.33	** < 0.001**
5	211	0.16	0.11–0.24	< 0.001	0.19	0.12–0.30	** < 0.001**
> 5	358	0.09	0.06–0.13	< 0.001	0.16	0.10–0.24	** < 0.001**
Missing*	91						
Diameter of largest CRLM in mm							** < 0.001**
< 20	1411	1			1		
20–34	1622	0.67	0.57–0.77	< 0.001	0.70	0.59–0.83	** < 0.001**
35–54	745	0.51	0.42–0.61	< 0.001	0.49	0.40–0.61	** < 0.001**
55–998	298	0.32	0.23–0.43	< 0.001	0.32	0.23–0.44	** < 0.001**
Missing	412	0.68	0.54–0.85	< 0.001	0.82	0.62–1.09	0.16
Bilobar disease				< 0.001			
No	2028	1			1		
Yes	1730	0.22	0.19–0.36	< 0.001	0.81	0.65–1.01	0.056
Missing	730	0.47	0.39–0.56	< 0.001	1.06	0.80–1.41	0.69
Location of primary tumour				0.001			
Colon	2905	1			1		
Rectal	1577	1.23	1.08–1.39		1.39	1.20–1.61	** < 0.001**
Missing*	6						
Timing of metastases				< 0.001			**0.007**
Metachronous	2412	1			1		
Synchronous	2076		0.68–0.87		1.23	1.06–1.43	
Missing*							
Extrahepatic disease				0.003			0.051
No	3806	1			1		
Yes	588	0.76	0.63–0.91		0.81	0.65–1.01	
Missing*	94						
Type of hospital**				< 0.001			** < 0.001**
Other hospitals	2573	1			1		
Tertiary centres	1951	0.50	0.44–0.57		0.57	0.50–0.67	

Bold p-values indicate statistical signficance (p < 0.05)

^*^Missing's where excluded from analyses when less than 5%

^**^Abbreviations: BMI indicates body mass index; ASA score indicates American Association of Anaesthesiologist; type of hospital, tertiary centre indicates hospitals with the highest expertise on oncological surgery

£ Histopathology of the liver on the basis of pathological examination in millimetre, other including: fibrosis, cirrhosis or sinusoidal dilatation

### Hospital and oncological network variation in use of minimally invasive liver resections

**Fig. 2:**
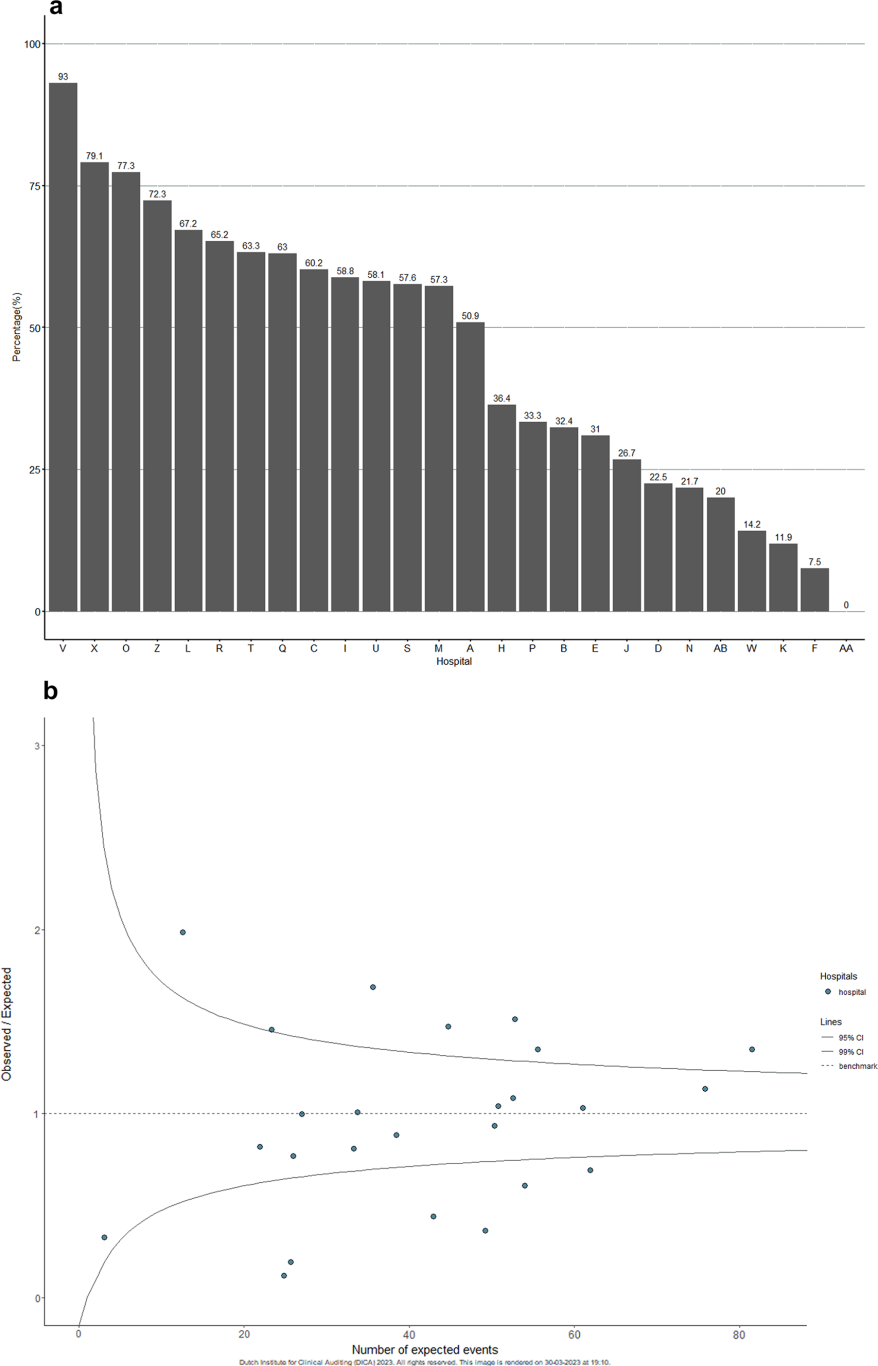
**a** Unadjusted hospital variation in use of MILR for minor resections for CRLM in the Netherlands between 2018–2021. **b** Funnel plot of case-mix corrected hospital variation in use of MILR for minor liver resections in patients with CRLM in the Netherlands between 2018–2021. Observed/Expected: O/E ratio. Number of expected patients treated with MILR. Case-mix adjusted for age, Charlson Comorbidity Index (CCI) score, Body Mass Index (BMI), ASA score, number of colorectal liver metastases (CRLM), bilobar disease, liver disease, maximum diameter of largest CRLM, location of primary tumour, type of metastases, extrahepatic metastases and type of hospital

Variation was observed in the use of MILR between hospitals and oncological networks. Unadjusted rates of MILR between 2018 and 2021 ranged from 7.5% to 93.0% (Fig. 2A). All hospitals performing liver surgery used MILR. Based on case-mix factors, expected rates of MILR between 2018–2021 ranged from 29.2% to 64.7%. After correction for case-mix factors, six hospitals performed more MILR than expected, and six hospitals performed less MILR than expected (Fig. 2B).

For oncological networks, unadjusted rates of MILR between 2018 and 2021 ranged from 30.7% to 58.1% (Supplementary figure S3). After correction for case-mix factors, one oncological network performed less MILR than expected, and one network performed more MILR than expected (Supplementary figure S4).

Upward from 2020, distinction between minor resections for left lateral and anterior segments was possible. Hospital variation in the use of MILR for left lateral and anterior segments between hospitals was observed. Unadjusted rates ranged from 0.0% to 100.0%. After correction for case-mix factors, one hospital performed more MILR than expected, and four hospitals performed less MILR than expected (Supplementary figure S5).

### Propensity-score matching

After performing propensity score matching, 1338 patients were included in either group. Standardised mean differences (SMD) were < 0.10 for all patient and tumour characteristics, except for bilobar disease (62.3 vs 45.1%, *p* < *0.001*, SMD 0.36). Treatment characteristics were different for combined ablation (89.2% vs 80.1%, *p* < *0.001*), and treatment in tertiary referral centres (33.8% vs 43.2%, *p* < *0.001*) (Table 1).

### Postoperative outcomes

Multilevel multivariable logistic regression nested for the year of surgery in the PSM cohort showed that MILR was associated with less blood loss (200 ml vs 400 ml, OR 0.99 CI: 0.99–0.99, *p* < 0.01), less cardiac complications (1.1% vs 3.1%, OR 0.29, CI:0.10–0.70, *p* = 0.009), shorter hospital stay (4 days vs 6 days, 0.94–0.99, *p* < 0.01), and less postoperative IC admission (OR 0.66, CI: 0.50–0.89, *p* = 0.005) compared to OLR. Results are shown in Table 3 and Supplementary Table 1.

**Table 3:** Multilevel logistic regression model nested for year for perioperative outcomes for patients with colorectal liver metastases who underwent a minor liver resection between 2014 and 2021 in the Netherlands

Factor	Univariable				Multivariable		
	*N*	OR	95% CI	*p*-value	aOR	95% CI	*p*-value
Blood loss in ml				** < 0.001**			** < 0.001**
Median + IQR	300 (100–600)	0.99	(0.98–0.99)		0.99	0.99–0.99	
Missing	221						
Bile leakage				0.89			
No	2621	1					
Yes	41	1.04	0.55–1.94				
Missing	14						
Intra-abdominal infection				**0.003**			0.57
No	2443	1			1		
Yes	51	0.40	0.21–0.72		1.13	0.74–1.73	
Missing	182						
Surgical site infection				0.19			
No	2355	1					
Yes	144	0.80	0.56–1.12				
Missing	177						
Pneumonia				**0.009**			0.89
No	2393	1			1		
Yes	108	0.59	0.39–0.87		0.97	0.02–1.56	
Missing	175						
Cardiac complication				**0.01**			**0.009**
No	2600	1			1		
Yes	57	0.45	0.24–0.82		0.29	0.10–0.70	
Missing	19						
Overall–30-day morbidity				0.20			
No	2262	1					
Yes	414	0.87	0.70–1.07				
Missing	0						
30-day major morbidity				0.13			
No	2457	1					
Yes	219	0.81	0.61–1.06				
Missing							
30-day mortality				0.61			
No	2631	1					
Yes	16	1.28	0.47–3.61				
Missing	29						
Length of stay				** < 0.001**			** < 0.001**
Median + IQR	5 (3.00–7.00)	0.94	0.92–0.95		0.97	0.96–0.99	
Missing	33						
ICU admission				** < 0.001**			**0.005**
No	2254	1			1		
Yes	375	0.47	0.37–0.59		0.66	0.50–0.89	
Missing	47						
Resection margins				0.74			
R0	2403	1					
R1	220	1.03	0.78–1.35	0.82			
R2	17	0.69	0.25–1.81	0.46			
Missing	36						

### Overall survival

**Fig. 3:**
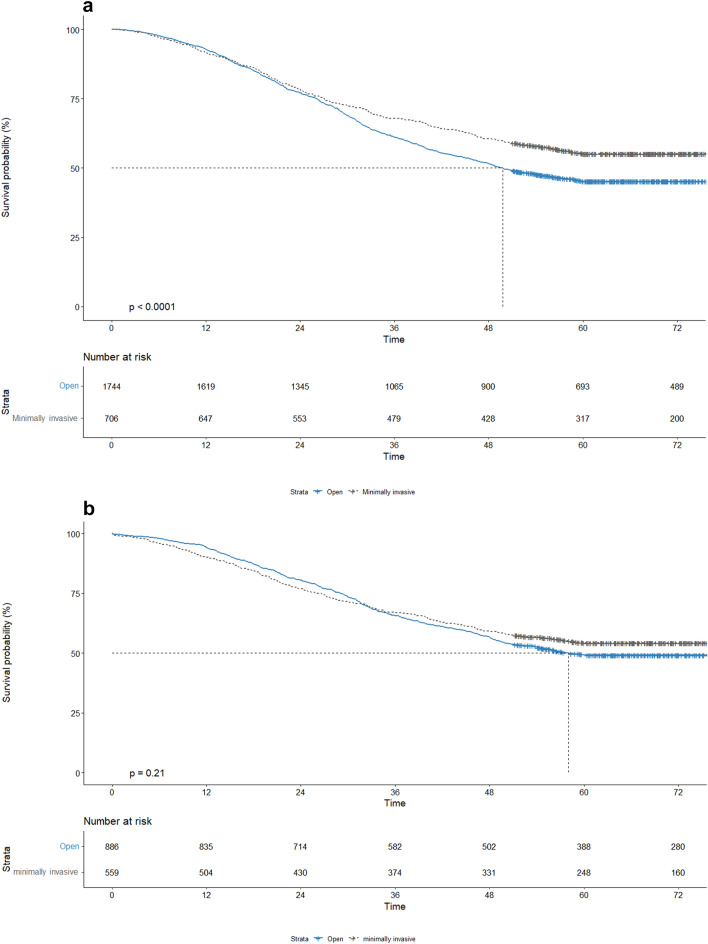
**a** Overall survival after minor open liver resection (blue) and minor minimally invasive liver resection (grey) before PSM in patients with colorectal liver metastases between 2014 and 2018. Time in days. **b** Overall survival after minor open liver resection (blue) and minor minimally invasive liver resection (grey) after PSM in patients with colorectal liver metastases between 2014 and 2018. Time in months (Color figure online)

Five-year OS for MILR versus OLR was 54.7% versus 44.9%, *p* < *0.01, i*n the overall cohort (Fig. 3A). In the propensity scored matched cohort, the median follow-up time was 69.9 (58.9–83.6) months. Five-year OS for MILR versus OLR was 53.7% versus 48.6%, *p* = 0.21 (Fig. 3B).

## Discussion

This nationwide population-based study evaluated the implementation, hospital variation, and outcomes after minor MILR in patients with CRLM in the Netherlands. The uptake of MILR for minor liver resections was originally slow but has increased in recent years. All liver surgery centres in the Netherlands had access to minimally invasive techniques. Remarkable hospital variation in the use of MILR remained. Short-term outcomes were better after MILR than OLR. No differences were observed in long-term survival.

This study reflected daily practice in the Netherlands and showed that implementation of MILR for minor liver resections is steadily progressing. This observation is consistent with data from other population-based studies that described implementation rates for minor MILR varying between 28.5% and 39.1% [15–18]. The implementation of minimally invasive liver surgery is still lagging behind other surgical procedures [19, 20].

This study showed relatively few factors which were negatively associated with the performance of MILR. These factors included maximum diameter and number of CRLM, preoperative chemotherapy, and treatment in tertiary referral centres. The first three are related as advanced disease is often treated with preoperative chemotherapy. Tertiary centres may perform liver resections in more complex cases, including advanced metastases in the liver, recurrent disease and patients with locally advanced colorectal tumours. Moreover, some centres, in particular some academic centres, have an active program for thermal ablation of liver metastases. From 2017 onwards, several studies have been ongoing in the Netherlands, including two trials (COLLISION and MAVERRIC) comparing thermal ablation to surgical resection in patients with resectable CRLM [21, 22]. Participation of centres in these trials could influence the use of MILR when patients eligible for these trials were treated with ablation alone, and patients not eligible (for example, due to tumour size, number, or a small distance to a major bile duct) could be more predisposed to undergo open liver surgery. Meanwhile, patients with low-risk CRLM were eligible for the PUMP trial in which resection was compared with resection plus adjuvant hepatic arterial infusion pump chemotherapy. Pump placement is often with an open approach [23]. Therefore, hospitals participating in this trial may perform more OLR, even in patients with metastases characterised as within Southampton guidelines. This influences the implementation rate of MILR.

Factors other than case-mix variables could contribute to significant variance in the implementation of MILR between hospitals. For example, the preferences and training of surgeons for a specific operative technique or use of thermal ablation may differ. Training programs for surgeons, such as LAELIVE and LIVEROBOT, could be implemented in all hospitals to improve the implementation of MILR [24]. However, to participate in these training programs, hospitals must have a sufficient volume of procedures. Not all hospitals meet this acquirement. Also, centralisation of liver surgery in the Netherlands created new referral patterns between hospitals. These referral patterns could increase practice variation due to lateralisation of care as more complex cases will be referred to tertiary or specialised centres and less complex cases to regional hospitals [25].

The superior short-term outcomes observed in this study, such as less blood loss, less postoperative complications, and shorter length of stay, are in line with randomised controlled trials such as the OSLO-COMET[26] and the LapOpHuva [27] and with several observational studies [28–32] and a recent meta-analysis [33]. This study confirmed that the two techniques showed comparable R0 resections and comparable overall survival in the matched cohorts. The same result was reported in several earlier cohort studies, but their retrospective data was predominantly derived from expert centres [28–31]. This real-world data study, including all liver surgery centres in the Netherlands, provides more decisive confirmation that when MILR is feasible, it is preferred over OLR. In the future, the aim is to minimise hospital variation in the use of MILR and increase adherence to the Southampton guideline.

Several limitations must be considered since the data was obtained from a nationwide retrospective database. Since long-term follow-up was not mandatory in the DHBA and the authors had to merge databases by personal citizen numbers, 7.4% of OS data was missing, which could have possibly biased OS results. Some patients, like soldiers and foreigners did not have these numbers. In addition, results may be biased since audit data did not contain specific and detailed information regarding perioperative variables such as proximity of hepatocaval veins, hilar vessels, or bile ducts, surgeon variation, and variation in the use of tumour ablation. Furthermore, several difficulty scores have been developed to specify the complexity of liver resections because not all parameters are registered in the DHBA and, therefore, unable to predict the complexity of performed liver resections correctly.

In conclusion, MILR is safe for minor liver resections and offers benefits regarding short-term outcomes without compromising survival. Significant hospital variation still exists in the Netherlands and should be reduced in the coming years.

## Supplementary Information

Below is the link to the electronic supplementary material.

Supplementary file1 (DOCX 15 kb)

Supplementary file5 (JPG 44 kb)

Supplementary file2 (JPG 39 kb)

Supplementary file3 (JPG 20 kb)

Supplementary file4 (JPG 20 kb)

Supplementary file6 (DOCX 14 kb)
