# Supplementary material for: Practice variation and outcomes of minimally invasive minor liver resections in patients with colorectal liver metastases: a population-based study
Source: Surg Endosc. 2023 Apr 18;37(8):5916–30. doi: 10.1007/s00464-023-10010-3 (PMC10338622; doi:10.1007/s00464-023-10010-3)
Supplement: Supplementary file 1 — Supplementary file1 (DOCX 15 kb) [file 464_2023_10010_MOESM1_ESM.docx]

**Table S1.** Cohort after matching. Postoperative outcomes of minor MILR and minor OLR for colorectal liver metastases between 2014-2021 in the Netherlands.

| **Table S1** |  |  | |
| --- | --- | --- | --- |
| Factor | MILR | OLR | p-value |
|  | N= 1338 | N = 1338 |  |
|  |  |  |  |
|  |  |  |  |
| **Blood loss in ml** |  |  | **<0.001** |
| Median + IQR | 200 (50.00 – 450) | 400 (200 – 750) |  |
| Missing | 99 | 122 |  |
|  |  |  |  |
| **Bile leakage** |  |  | **0.006** |
| No | 1316 (98.4) | 1305 (97.5) |  |
| Yes | 21 (1.6) | 20 (1.5) |  |
| Missing | 1 (0.1) | 13 (1.0) |  |
|  |  |  |  |
| **Intra-abdominal infection** |  |  | 0.14 |
| No |  |  |  |
| Yes | 65 (4.9) | 79 (5.9) |  |
| Missing |  |  |  |
|  |  |  |  |
| **Surgical site infection** |  |  | **0.003** |
| No | 1242 (92.8) | 1201 (89.9) |  |
| Yes | 15 (1.1) | 36 (2.7) |  |
| Missing | 81 (6.1) | 101 (7.5) |  |
|  |  |  |  |
| **Pneumonia** |  |  | **0.01** |
| No | 1217 (91.0) | 1176 (87.9) |  |
| Yes | 41 (3.1) | 67 (5.0) |  |
| Missing | 95 (7.1) | 80 (6.0) |  |
|  |  |  |  |
| **Cardiac complication** |  |  | **<0.001** |
| No | 1320 (98.7) | 1280 (95.7) |  |
| Yes | 15 (1.1) | 42 (3.1) |  |
| Missing | 3 (0.2) | 16 (1.2) |  |
|  |  |  |  |
| **Overall – 30-day morbidity** |  |  | 0.86 |
| No | 1143 (85.4) | 1119 (83.6) |  |
| Yes | 195 (14.6) | 219 (16.4) |  |
| Missing |  |  |  |
|  |  |  |  |
| **30-day major morbidity** |  |  | 0.15 |
| No | 1239 (92.6) | 1218 (91.0) |  |
| Yes | 99 (7.4) | 120 (9.0) |  |
| Missing |  |  |  |
|  |  |  |  |
| **30 – day mortality** |  |  | 0.86 |
| No | 1314 (98.2) | 1317 (98.4) |  |
| Yes | 9 (0.7) | 7 (0.5) |  |
| Missing | 15 (1.1) | 14 (1.0) |  |
|  |  |  |  |
| **Length of stay in days** |  |  | **<0.001** |
| Median + IQR | 4.00 (2.00 – 6.00) | 6.00 (5.00 – 9.00) |  |
| Missing | 14 | 19 |  |
|  |  |  |  |
| **ICU admission** |  |  | **<0.001** |
| No | 1195 (89.3) | 1059 (79.1) |  |
| Yes | 131 (9.8) | 244 (18.2) |  |
| Missing | 12 (0.9) | 35 (2.6) |  |
|  |  |  |  |
