# Supplementary figures and images for: Practice variation and outcomes of minimally invasive minor liver resections in patients with colorectal liver metastases: a population-based study
Source: Surg Endosc. 2023 Apr 18;37(8):5916–30. doi: 10.1007/s00464-023-10010-3 (PMC10338622; doi:10.1007/s00464-023-10010-3)

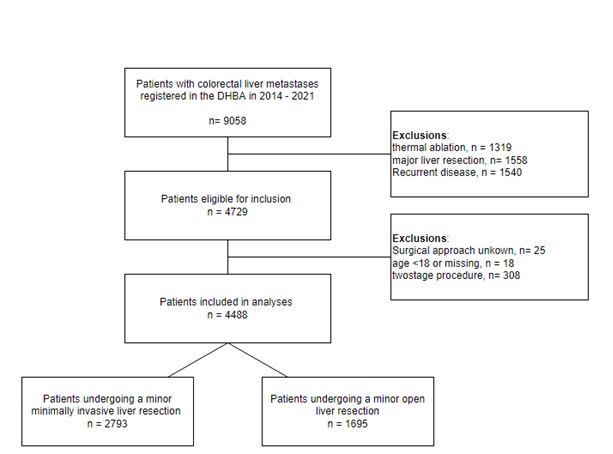

Supplement: Supplementary file 2 — Supplementary file5 (JPG 44 kb) [file 464_2023_10010_MOESM2_ESM.jpg]

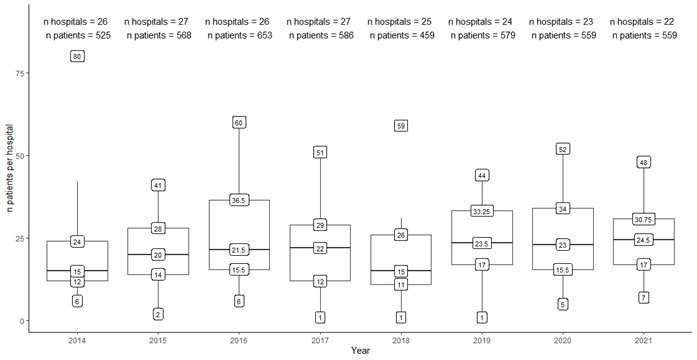

Supplement: Supplementary file 3 — Supplementary file2 (JPG 39 kb) [file 464_2023_10010_MOESM3_ESM.jpg]

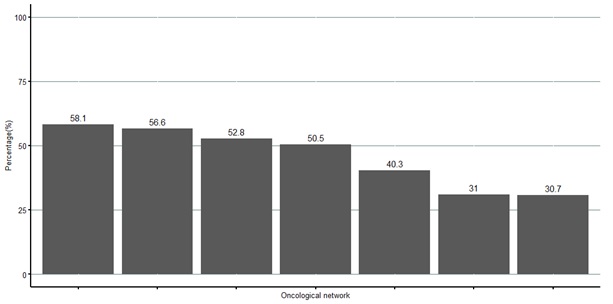

Supplement: Supplementary file 4 — Supplementary file3 (JPG 20 kb) [file 464_2023_10010_MOESM4_ESM.jpg]

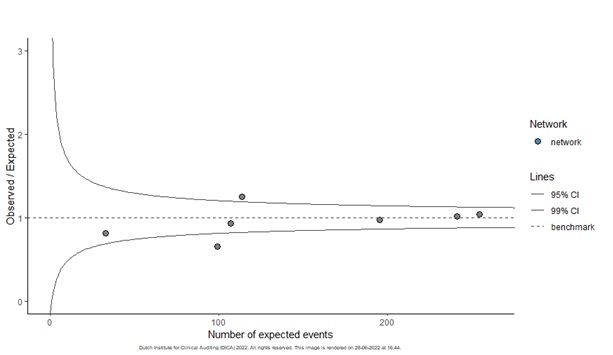

Supplement: Supplementary file 5 — Supplementary file4 (JPG 20 kb) [file 464_2023_10010_MOESM5_ESM.jpg]
