# Supplementary material for: Practice variation and outcomes of minimally invasive minor liver resections in patients with colorectal liver metastases: a population-based study
Source: Surg Endosc. 2023 Apr 18;37(8):5916–30. doi: 10.1007/s00464-023-10010-3 (PMC10338622; doi:10.1007/s00464-023-10010-3)
Supplement: Supplementary file 6 — Supplementary file6 (DOCX 14 kb) [file 464_2023_10010_MOESM6_ESM.docx]

**Supplementary figures**

**Figure S1**. Flowchart of the study.

**Figure S2**: Annual number of hospitals performing minor liver resections and volume of patients who underwent minor liver surgery. The boxplot represents the median number of registered patients per hospital with the interquartile and total range.

**Figure S3**. Unadjusted oncological network variation in use of MILR for minor resections for CRLM in the Netherlands between 2018 – 2021.

**Figure S4.** Funnel plot of case-mix corrected hospital variation in use of MILR minor resections in patients with CRLM in the Netherlands between 2018-2021. Observed/Expected: O/E ratio. Number of expected patients treated with MILR. Case-mix adjusted for: age, Charlson Comorbidity Index (CCI) score, Body Mass Index (BMI), ASA score, number of colorectal liver metastases (CRLM), bilobar disease, liver disease, maximum diameter of largest CRLM in millimetres, location of primary tumour, type of metastases, extrahepatic metastases and type of hospital.
